# Compartmentation of sucrose during radial transfer in mature sorghum culm

**DOI:** 10.1186/1471-2229-7-33

**Published:** 2007-06-20

**Authors:** Lee Tarpley, Donald M Vietor

**Affiliations:** 1 Texas A&M Agricultural Research and Extension Center, 1509 Aggie Dr., Beaumont, TX 77713, USA; 2 Department of Soil and Crop Sciences, Texas A&M University, College Station, TX, USA

## Abstract

**Background:**

The sucrose that accumulates in the culm of sorghum (*Sorghum bicolor *(L.) Moench) and other large tropical andropogonoid grasses can be of commercial value, and can buffer assimilate supply during development. Previous study conducted with intact plants showed that sucrose can be radially transferred to the intracellular compartment of mature ripening sorghum internode without being hydrolysed. In this study, culm-infused radiolabelled sucrose was traced between cellular compartments and among related metabolites to determine if the compartmental path of sucrose during radial transfer in culm tissue was symplasmic or included an apoplasmic step. This transfer path was evaluated for elongating and ripening culm tissue of intact plants of two semidwarf grain sorghums. The metabolic path in elongating internode tissue was also evaluated.

**Results:**

On the day after culm infusion of the tracer sucrose, the specific radioactivity of sucrose recovered from the intracellular compartment of growing axillary-branch tissue was greater (nearly twice) than that in the free space, indicating that sucrose was preferentially transferred through symplasmic routes. In contrast, the sucrose specific radioactivity in the intracellular compartment of the mature (ripening) culm tissue was probably less (about 3/4's) than that in free space indicating that sucrose was preferentially transferred through routes that included an apoplasmic step. In growing internodes of the axillary branch of sorghum, the tritium label initially provided in the fructose moiety of sucrose molecules was largely (81%) recovered in the fructose moiety, indicating that a large portion of sucrose molecules is not hydrolysed and resynthesized during radial transfer.

**Conclusion:**

During radial transfer of sucrose in ripening internodes of intact sorghum plants, much of the sucrose is transferred intact (without hydrolysis and resynthesis) and primarily through a path that includes an apoplasmic step. In contrast, much of the sucrose is transferred through a symplasmic path in growing internode (axillary branch) tissue. These results contrast with the probable symplasmic path in mature culm of the closely related species, sugarcane. Phylogenetic variability exists in the compartmental path of radial transfer of sucrose in culms of the andropogonoid grasses.

## Background

The Andropogoneae tribe of grasses includes a number of large tropical grasses, several of which are widely cultivated for either their grain (sorghum [*Sorghum bicolor *(L.) Moench] and maize [*Zea mays *L.]) or for the sucrose accumulated in the culm (sugarcane [*Saccharum officinarum *L.] and sorghum). The examined species contain sucrose in the culms. This sucrose can support grain filling in some circumstances by buffering the supply of photoassimilate. This study contributes to improved understanding of processes of sucrose accumulation in sorghum culm. Our first objective investigated whether radial transfer of sucrose from phloem to the storage compartment of ripening culm (elongated internode during sucrose accumulation) included an apoplasmic step. Second, we evaluated the metabolic path of sucrose, including the extent of sucrose hydrolysis, during this radial transfer within a growing culm (axillary branch). These results complement those of a previous study [1] that indicated much of the sucrose is not hydrolyzed during radial transfer in ripening culm.

Sorghum is closely related to sugarcane [2]; members of both species are capable of accumulating large amounts of sucrose in the culms. Similarities exist between the two species with respect to the processes of sucrose accumulation in ripening internodes. For both, a low level of sucrose degradative activity, especially acid invertase activity relative to sucrose synthetic activity, is a prerequisite for sucrose accumulation [3-6]. A large proportion of the sucrose is neither degraded nor synthesized during radial transfer in ripening sorghum internodes [1]. Sucrose can also be transferred intact in sugarcane internodes [7,8], but sucrose synthetic activity further promotes the sucrose accumulation [5].

The radial transfer of sucrose follows a symplasmic path in sugarcane [9,10] based on evidence from selective movement of compartmental tracer dyes and of lignification and suberization situated to prevent apoplasmic movement of sucrose between the vascular bundles and parenchyma in the ripening sugarcane internodes. Other evidence for systematic blockage of plasmodesmata suggests that an apoplasmic step might be necessary in radial transfer in sugarcane [11].

In internodes of mature sorghum culm, the sucrose moves along its metabolic path during radial transfer without requiring hydrolysis. This conclusion is based on several points of evidence. The extractable activities of the sucrose degradative enzymes, invertase (EC 3.2.1.26) and sucrose synthase (EC 2.4.1.13), decline to low levels prior to the period of extensive sucrose accumulation in diverse sorghums representing a range of culm sucrose-accumulating potential [3,12,13]. This was confirmed in the case of sucrose synthase by a concurrent decline in the levels of transcript for the sucrose-synthase gene [3]. In addition, sucrose is found at much higher concentration than either glucose or fructose in both the free space and the intracellular compartment of culms during storage [14]. Lastly, sucrose recovered from the intracellular compartment of a distant part of culm after infusion of asymmetrically labelled sucrose did not possess much randomization of label. Extensive randomization of label would be expected if the sucrose had been hydrolyzed and resynthesized [1]. In the same study, the sucrose appeared to preferentially move in a cellular path that included an apoplasmic step. This preliminary conclusion was based on a higher sucrose specific radioactivity being present in the free space relative to the intracellular compartment of the sampled culm tissue. Endogenous sucrose would dilute the radioactive sucrose, thus a higher sucrose radioactivity indicates a preferential or prior movement through that compartment. The radial transfer processes of sucrose into intracellular storage in the culms of sorghum delimit the processes of remobilisation from culm to grain, thus identifying the sucrose-accumulation processes also helps refine targets for improving or stabilizing sorghum grain yield.

As sorghum internode elongation nears completion, sucrose accumulates in the culm tissue [12]. The rate of accumulation in culm internodes often increases near anthesis, which is typically a period of low demand in reproductive sinks of uniculm sorghum types selected and managed for grain production [15]. Sucrose accumulated in culm tissue during anthesis can be remobilized to reproductive sinks if photoassimilate export from source leaves is low relative to reproductive sink demand [15,16]. Conversely, sucrose accumulates in mature internodes as grain filling is completed before new axillary branches develop from upper nodes of many of the non-senescent, tropically adapted sorghums [17]. The emergence of axillary branches after grain filling is complete offers an opportunity to compare, simultaneously, the processes of sucrose radial transfer from phloem to the intracellular storage/utilization sites in mature internodes of the main culm and in elongating internodes of rapidly growing branches. The cellular and metabolic paths of sucrose during radial transfer between phloem and the intracellular compartment in growing sorghum internodes have not been studied in intact plants, but would be expected to be symplasmic and without extensive sucrose hydrolysis based on comparison with other growing plant tissues [18].

The primary objective of this study was to determine if radial transfer of sucrose between phloem and the intracellular compartment in ripening internodes of sorghum included passage through an apoplasmic step. Radiolabelled sucrose was introduced into culms of two sorghum cultivars; the sucrose recovered from unperturbed tissue for analysis; and the sucrose specific radioactivity compared between apoplasmic (free space) and the intracellular compartment. Because endogenous sucrose dilutes radiolabelled sucrose, the comparative dilution of sucrose specific radioactivity is indicative of the compartmental path during radial transfer.

A secondary objective was to compare the radial path of sucrose from phloem to intracellular compartment of growing axillary-branch tissue with that of the ripening internode tissue. A previous study indicated much of the sucrose was not hydrolyzed and resynthesized during radial transfer in ripening sorghum internode, but the metabolic path in elongating internode tissue was not examined. Asymmetrically labelled ^3^H-sucrose (label in the fructose moiety only) was infused into culms, sucrose recovered from the intracellular compartment of unperturbed branch tissue was analyzed, and the distribution of ^3^H-label between the two hexose moieties of sucrose was quantified. An extensive amount of redistribution of label between the moieties would indicate that extensive hydrolysis and resynthesis of sucrose occurred during the radial transfer, whereas little redistribution of radiolabel in the recovered sucrose molecules would indicate that little hydrolysis and resynthesis had occurred.

## Results

### No gradient of radiolabel from tracer sucrose along the length of the sampled internode

Radiolabel content was similar among sampling locations of ripening internode tissue. These locations were opposite and below the infusion point. The uniform radiolabel content among the locations indicated neither tissue damage nor simple diffusion through culm tissue could explain the movement of radiolabeled sucrose into the sampled portion of internode. Instead, the lack of a gradient in the radiolabel along the length of the sampled portion of the internode is consistent with delivery of the radiolabelled sucrose through phloem to the sampled portion of the internode. Similar results were obtained in previous studies [1,19,20], and the delivery of the radiotracer through normal distribution routes (i.e., phloem) was a basic assumption of the methodology used in this study. The lack of variation of radiolabel content among sampling locations indicated the 24 samples analyzed in the present study provided an unbiased representation of a) internode location, b) cultivar, c) developmental stage, and d) intracellular compartment.

### Sucrose and hexose-sugar contents of intracellular and free-space compartments

**Figure 1:**
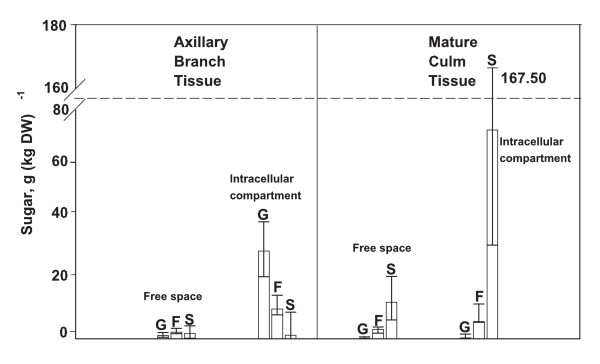
**Soluble sugars of free-space and intracellular compartment in elongating and ripening internode of sorghum**. The contents of glucose (G), fructose (F) and sucrose (S), expressed on a weight per tissue weight basis, of the free space (left subpanel) and the intracellular compartment (right subpanel) of elongating internode tissue from axillary branches at or before anthesis (left panel) and of mature ripening internode tissue of the main culm (right panel) of sorghum. The results from two cultivars of sorghum are combined. The error bars are 95% confidence intervals.

Culm soluble sugars were located largely within the intracellular compartment of both ripening and elongating internodes. The mean percentage of soluble sugars in the intracellular compartment was 83% for ripening internodes of the main culm and 89% for elongating internodes of axillary branches (Fig. 1). The volumes of the intracellular compartment and free space were not determined, which made it necessary to compare relative contents rather than concentrations of sugar. Total soluble-sugar (glucose + fructose + sucrose) contents, normalized on a tissue dry weight basis, were greater in both compartments of the ripening-culm tissue than in respective compartments of the elongating-internode tissue (results not shown). A more striking difference was the greater ratio of sucrose to hexose (glucose + fructose) sugar (> 1) for both compartments of the ripening-culm tissue than for respective compartments of elongating-internode tissue (< 1) (Fig. 1).

### ^14^C-sucrose specific radioactivity of intracellular and free-space compartments

**Figure 2:**
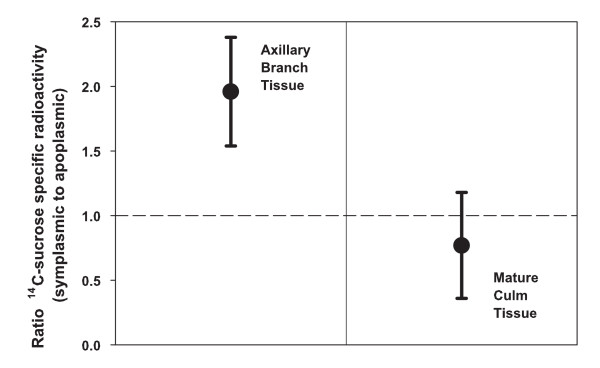
**Ratio of the ^14^C-sucrose specific radioactivity of the intracellular compartment relative to free space**. The ratio of the ^14^C-sucrose specific radioactivity of the intracellular compartment relative to the free space of elongating internode tissue from axillary branches at or before anthesis (left panel) and of ripening internode tissue of the main culm (right panel) of sorghum. Radiolabeled sucrose had been introduced into intact plants via culm infusion about 24 h previously. The reference line is at a ratio of 1.0. The results from two sorghum cultivars are combined. The error bars are 95% confidence intervals.

The ratio of the ^14^C-sucrose specific-radioactivity in intracellular compared to free-space compartments (sucrose specific-radioactivity ratio) was calculated to determine, in part, the path of sucrose during transfer between phloem and intracellular compartments. The ^14^C-sucrose specific radioactivity is the ^14^C-radioactivity recovered as sucrose from a compartment divided by sucrose content of the same compartment. As infused radiolabelled sucrose moves throughout the plant, the sucrose specific radioactivity decreases due to dilution from the endogenous sucrose. A relatively high sucrose specific-radioactivity ratio would indicate the radial path of the sucrose is primarily intracellular (symplasmic). A ratio greater than 1 (1.96 ± 0.42 [95% c.i.]) in elongating internodes of axillary branches (Fig. 2) indicated sucrose is preferentially moved into the intracellular compartment through symplasmic routes.

In the ripening internode of the main culm, the sucrose specific-radioactivity ratio was less than in the elongating internode and probably less than 1 (0.77 ± 0.41 [95% c.i.]) (Fig. 2). The preferential path of sucrose radial transfer of ripening internodes is likely to include an apoplasmic step. Similar results of a previous study [1] indicated the path in the ripening internode included an apoplasmic step. In combination, the results from the two studies provide strong evidence that the preferential route in the ripening stem includes an apoplasmic step.

### Radioactivity distribution between hexose moieties of sucrose recovered from intracellular compartment of elongating internodes

Pearson's correlation analysis was used to compare the sucrose specific-radioactivity ratio between ^14^C- and ^3^H-sucrose that were infused simultaneously and sampled from elongating internodes of the axillary tillers. The correlation coefficient was 0.988. The strong positive correlation indicated both ^14^C- and ^3^H-sucrose were quantitatively similarly transported into the intracellular compartment of elongating internodes of sorghum. These results suggest that isotope discrimination during radial transfer of sucrose had little influence on the redistribution of ^3^H-label between the hexose moieties of sucrose as an indicator of the metabolic path of the sucrose.

**Figure 3:**
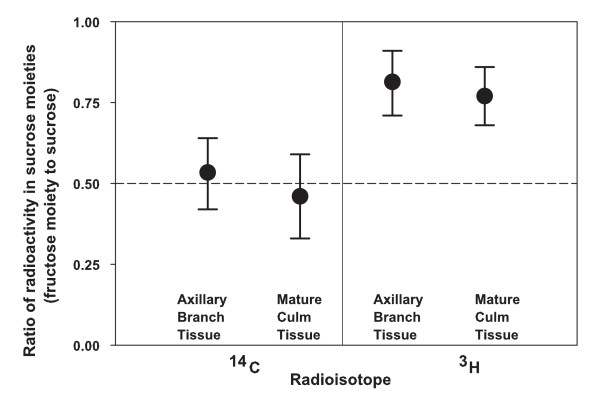
**Proportion of radioactivity in fructose moiety of sucrose recovered from intracellular compartment of internode tissue**. The proportion of ^14^C (left panel) and ^3^H (right panel) in the fructose moiety relative to the combined fructose and glucose moieties of sucrose recovered from the intracellular compartment of internode tissue. Tissue was sampled from unperturbed internode tissue of sorghum plants into which uniformly labelled ^14^C-sucrose and asymmetrically labelled (all of the label in the fructose moiety) ^3^H-sucrose had been simultaneously infused about 24 h before. The proportions are provided for tissue from axillary branches at or before anthesis and for mature ripening tissue from the main culm. The results for the main culm are from a previous study [1]. In each case, the results from two sorghum cultivars are combined. The reference line is at a proportion of 0.5, which was the proportion of the ^14^C-sucrose at the time of introduction into the plant. The error bars are 95% confidence intervals.

Upon introduction into the plants, the ^3^H-sucrose contained all of the ^3^H-label in the fructose moiety. If the sucrose was hydrolyzed (inverted) and resynthesized along the route to the intracellular storage compartment, ^3^H-label would be redistributed between the two hexose moieties of the resynthesized sucrose because of the omnipresence and activity of phosphoglucoisomerase (EC 5.3.1.9) [1]. The equal distribution of ^14^C-label between the two hexose moieties of ^14^C-sucrose provided an unchanging reference for the distribution of radioactivity between hexose moieties. The ^14^C-radioactivity recovered in sucrose of the intracellular compartment of the axillary-branch tissue remained equally distributed between the two hexose moieties (Fig. 3) as observed previously for ripening sorghum internodes [1] (Fig. 3). In contrast, the ^3^H-label in the recovered sucrose remained in the fructose moiety (81%) of elongating internodes (Fig. 3). This same result was observed for ripening sorghum internodes in the previous study [1] (Fig. 3). Assuming a Poisson distribution, a large portion (52% ± 33% [95% c.i.]) of the fructose moiety of sucrose was not exposed to hydrolysis (i.e., sucrose cleavage, hexose phosphorylation, isomerization of hexose phosphates, and sucrose resynthesis) during radial transfer of sucrose in the elongating internodes of the growing axillary-branches of intact sorghum plants [7].

## Discussion

### Soluble sugar within the intracellular compartment of the internodal tissue

The larger sucrose to hexose-sugar ratio observed in ripening internode tissue relative to elongating internode tissue (Fig. 1) is consistent with variation in soluble-sugar composition during maturation of sorghum culms [1,3,12]. Previous studies indicated the increase in sucrose relative to hexose sugar is preceded by a decline in sucrose-degradative activity during the transition from internode elongation to the ripening phase [3,12]. The high content of soluble sugars in the intracellular compartment of internodes of axillary branches and the ripening internodes (Fig. 1) is substantiated in previous reports [1,3]. When plant tissues contain high levels of osmoticum, including soluble sugars, the osmoticum concentrations of the free space and intracellular compartment are fairly comparable [18]. In the present study, the higher content of soluble sugars in the intracellular compartment reflects, in part, the greater volume compared to free space.

The relatively high ratio of glucose to fructose in the intracellular compartment of the axillary branch is probably a result of greater consumption of fructose. The procedures for sugar analysis were modified from ones known to prevent artifactually high fructose levels in plant tissue extracts [21]. The starch levels in sorghum internodes are typically quite low [3], so do not sequester enough glucose to explain the high ratio as due to high glucose influx. In asparagus stem, a greater consumption of fructose was related to increased capacity for glycolytic flux in the stem [22]. However, the transition from a high glucose to fructose ratio as noted in immature grape berries to one near unity in mature grape berries was associated with a breakdown of cell integrity during ripening resulting in a loss of the spatial separation of substrate and enzyme (invertase) [23]. In mature sugarcane internode, the fructose is thought to be maintained at low levels due to high fructose phosphorylating activity [24].

### Apoplasmic step in path of radial transfer of sucrose in ripening internode

In the growing axillary branch of sorghum, the preferential route of sucrose during radial transfer from phloem to the intracellular compartment is symplasmic (Fig. 2). A large portion of sucrose is transferred intact (Fig. 3) without hydrolysis or resynthesis. In ripening internodes of sorghum, the preferential route of sucrose during radial transfer includes passage through an apoplasmic compartment (Fig. 2). Yet, a large portion of the sucrose is transferred intact (Fig. 3).

The symplasmic transfer of the intact sucrose to the growing internodes of the axillary branch is consistent with expectations for paths in growing plant organs [25]. There is no obvious advantage for an apoplasmic step within the steep concentration gradient between phloem and growing cells and tissue. The sucrose moves into growing cells and has a relatively short half-life before being degraded and used for various purposes [26]. The utilization of sucrose in the cell maintains a concentration gradient allowing more sucrose to enter into the intracellular storage compartment via diffusion, bulk flow, or regulated plasmodesmatal conductance [26].

The presence of an apoplasmic step during radial transfer of sucrose in ripening internodes of sorghum is consistent with expectations for plant organs after the transition from growth to accumulation of large amounts of osmoticum [18]. The apoplasmic step can enable regulation of cellular uptake of sucrose at the membrane-transporter level. However, the route in the ripening internode in sorghum differs from that in sugarcane, a closely related species, which appears to involve symplasmic transfer [9].

A positive association between the extent of competition between parenchymal uptake and phloem uptake of sucrose and the sucrose concentrating ability of sorghum internodal tissue from different genotypes and developmental stages [19], in combination with evidence for radial transfer of sucrose intact [1] and through an apoplasmic compartment, suggests a possible role for a sucrose transporter in the plasmalemma or tonoplast membrane as part of a mechanism regulating sucrose accumulation in sorghum culm, but no direct evidence exists in support of this. Furthermore, there is no evidence to rule out a role for hexose transport from the apoplasm, but under the conditions of the present study this would be a secondary role. In sugarcane, the ShSUT1 sucrose transporter is localized in parenchyma tissue surrounding the stem vascular bundles [10] where it might be involved in sucrose efflux to the apoplasm or in return of apoplasmic sucrose to the symplasm. In the latter case, the sucrose transporter might be acting in conjunction with the lignified, suberized cell walls surrounding the vascular bundles to provide both a biochemical and a physical barrier against backflow of sucrose into the vascular system [27]. Evidence exists for uptake of sucrose intact [7,8] into sugarcane internode tissue, and also for hexose uptake into suspension cells derived from sugarcane culm parenchyma tissue [28]. Hexose transporters have been identified in sugarcane internodes with expression in the vascular bundle [29] and in the surrounding parenchyma cells [27]. The mechanisms for tonoplast uptake of sucrose in sugarcane internode are not clear [27]. The expression patterns of sugar transporters in sugarcane culm do not exclude any of the suggested cellular or metabolic paths of sucrose during radial transfer.

Although the transcriptome has been analyzed for various sorghum tissues, developmental stages and environmental conditions (e.g. [30]), the objectives of these studies have not included the provision of information to help define the mechanisms of sucrose accumulation in ripening sorghum internode [31]. A number of putative sugar transporters have been identified via transcriptomic studies in sugarcane [29,31,32]. These, but especially the ShSUT1, have potential as probes for examining the expression patterns in sorghum internodes and providing insight into the mechanisms of sucrose accumulation in sorghum culm.

Sucrose synthetic metabolism in ripening internodes of sugarcane might be sufficient to balance sucrose degradative activity remaining during the transition from internode elongation to ripening [5]. If the sucrose synthetic metabolism of sugarcane does have this effect of increasing the sink strength of the ripening internode relative to that of sorghum, then it might allow an earlier and greater accumulation of sucrose. On the other hand, it could also diminish the ability to remobilize sucrose from the ripening culm tissue.

### Intact transfer of sucrose from phloem to intracellular compartments

Observations of radial transfer of asymmetrically labeled (^3^H) sucrose to the elongating internodes of sorghum axillary branches indicate that there is no requirement for hydrolysis and resynthesis of sucrose. Similarly, a substantial portion of sucrose is transported intact between phloem and intracellular compartments within ripening sorghum culm internodes. The transport of intact sucrose agrees with recent models from sorghum [1] and sugarcane [7], which do not include a path involving invertase action that cleaves sucrose prior to hexose phosphorylation, isomerization of hexose phosphates, and sucrose resynthesis during sucrose uptake into the intracellular compartment. The isomerization of the hexose phosphates due to phosphoglucoisomerase action is usually rapid and easily reversible. The unchanged asymmetry of ^3^H-label between hexose moieties of sucrose transported to elongating internodes in the present study indicates inversion of sucrose and isomerization of hexoses is not necessary for radial transfer of sucrose between phloem and intracellular compartments.

Previous studies to determine the metabolic path of sucrose have often involved application of the tracer sucrose to slices of culm tissue (stem disks). The initial efforts supporting the inversion model, which hypothesized sucrose hydrolysis and resynthesis [33], involved incubation or washing of the tissue slices for extended periods in solution lacking osmoticum before sucrose uptake was quantified. The steep concentration gradient between wash solutions and culm tissue could have increased cell turgor and induced invertase activity. The invertase activity would enable sucrose hydrolysis prior to sucrose accumulation in cells within disks [34]. The interpretation of observations of sucrose uptake in stem disks (portions of culm cross-sections) from solution presents additional challenges. Sectioning and suspension of disks in solution can interrupt or bypass normal anatomical flow of sucrose from phloem to the intracellular compartment. Rapid depletion of the sucrose pool of sieve elements and companion cells could result due to interruption of normal routes of replenishment [35]. In more recent studies, tissue slices from ripening sugarcane internodes were incubated in osmotically buffered solution to avoid the steep turgor gradients during washing in early studies. Uptake of asymmetrically labeled sucrose from the osmotically buffered solutions provided preliminary evidence that sucrose was not necessarily cleaved and resynthesized in route to storage in cells [7,8]. These more recent studies of sucrose uptake warned against uncritical acceptance of the inversion model, but the inherent limitations of experiments using excised disks in solution equivocated arguments against the inversion model, and also could not determine the compartmental path of the sucrose during radial transfer. The results from intact sorghum internodes in the present study show that the inversion model is not universally valid for the andropogonoid grasses.

Evidence that a substantial portion of the sucrose is transferred intact from the phloem to the storage compartment in sorghum culm does not rule out the possibility that sucrose can also be inverted in the apoplasmic space followed by cellular uptake of the hexose sugars. Evidence for such uptake exists in sugarcane internode cells [28]. The ratios of these two sucrose metabolic paths during radial transfer in sorghum internode under various conditions has not been studied.

### Nature of sink strength during sucrose accumulation in ripening sorghum internode

'In planta' and molecular studies similarly indicate sucrose metabolism is not necessary for sucrose storage in ripening sorghum internodes. Extractable activities of sucrose-degrading enzymes decline to low levels in internodes prior to sucrose accumulation [3,12]. The decline includes repression of activity at the pretranslational level [3]. Yet, relatively low levels of sucrose degradation do not imply that the ripening sorghum internode is a passive sink. A previous culm infusion study performed on sorghum [19] showed that the gradient in radiolabel content directly below the infusion site was related to potential sink strength. Cellular uptake in internode tissue was greater in a sweet-stemmed than a grain-type cultivar. In addition, competition between phloem removal and intracellular storage of provided sucrose was greater for the sweet-stemmed cultivar. The preceding decline in activities of the sucrose-degrading enzymes, however, did not relate to potential sink strength. The results from the present study indicate radial transfer of intact sucrose in ripening internodes is compatible with a mechanism of sucrose accumulation that includes regulation of cellular uptake. The mechanism in sorghum internodes regulating sucrose accumulation and remobilization is likely to lie in the combination of regulation of cellular uptake of sucrose at the membrane transporter level, passive resistance to back-flow of the sucrose due to distance between some storage cells and phloem, and/or regulation of flow through the plasmodesmata. These possibilities were not addressed in this study.

## Conclusion

In the growing axillary branch of sorghum, the preferential cellular path of sucrose during radial transfer from phloem to the intracellular compartment is symplasmic, and much of the sucrose is transferred intact. In the mature ripening internode, the compartmental path preferentially includes an apoplasmic step, and much of the sucrose is transferred intact. Phylogenetic variation in the extent to which an apoplasmic step is involved in the radial transfer of sucrose in culms of the large tropical grasses of the Andropogoneae needs to be defined, instead of extrapolating sugarcane-based models of accumulation to other species. The presented method of introducing tracer sucrose into intact plants via culm infusion avoids difficulties in interpretation of results obtained through use of tissue slices.

## Methods

### Plant material and culture

Plants of two semidwarf grain sorghum types (Tx430 and ATx631 X RTx436) were grown in research field plots using typical production practices at the Texas A&M University Research Farm in Burleson County, Texas, USA. The plants were healthy at the time of treatment.

### Culm infusion

Infusion of radiolabeled sucrose into internodes of intact plants was used to trace radial paths of transport between phloem and intracellular compartments. The culm infusion of sucrose solution was achieved for six plants of each cultivar during a developmental period in which at least one axillary branch (an axillary branch develops from an upper node, typically no earlier than late grain-filling of the main panicle) was at or nearing anthesis. At this developmental stage, the upper internodes of the axillary branch are still elongating, but internodes on the main culm are fully elongated. The sites for infusion on the main culm were about ten internodes above soil level, which avoided short basal internodes close to root sinks. In addition, infusion sites were separated by about three internodes from sink effects of the developing panicle and axillary branches. Because of these compromises, the infusion internode was also chosen for analysis of mature culm tissue. Previous study had demonstrated that the portion of an elongated sorghum internode below and opposite the infusion site is not subject to injury due to the infusion. Sampling to avoid injured tissue avoided diffusion or mass flow of radiolabeled sucrose between the infusion site and sampled tissue [19]. The spread of necrosis resulting from culm infusion in maize was no farther than 1 cm from the infusion cavity [28]. The wound response due to infusion of sweet sorghum extended no farther than 15 to 30 mm from the infusion cavity based on the ratio of radiolabel from the infused sucrose appearing in aqueous-ethanol insoluble relative to aqueous-ethanol soluble fractions of tissue extracts [19]. The mature culm samples were obtained at distances ranging from 40 to 70 mm from the infusion cavity. The samples from axillary branches were much farther from the infusion site, typically one to three internodes plus some portion of the branch distant.

The infusion into the selected culm internode was performed over a 1-h period in mid-afternoon as described in Tarpley et al. (1996) [1]. The general method of culm infusion used in the study was described by Boyle et al. [36]. A cork borer was used to drill a cylindrical hole (about 0.2 mL in capacity) partway into an upper portion of an internode. The cavity was then plugged with a serum sleeve stopper. After creating the cavity and before plugging it, the cavity was immediately filled with unlabeled sucrose solution to limit the amount of air introduced into the tissue while working the stopper into place. A small reservoir fed solution through tubing to a hypodermic needle that was inserted through the stopper into the cavity. About 400 μL was infused through tubing: first, 100 μL as unlabeled solution to make sure uptake was occurring; next, 100 μL containing labeled sucrose; and finally, 200 μL as a rinse with unlabeled solution. Complete introduction of label into culm was achieved for every plant. After infusion of the 204 mM (68 g L^-1^) sucrose solution containing 148 kBq (4 μCi) [U-^14^C]-sucrose (ICN Biomedicals, Irvine, California, USA) and 296 kBq (8 μCi) [fructose-1-^3^H(N)]-sucrose (Sigma Chemical Co., St. Louis, Missouri, USA), the needle feeding the cavity was removed.

Plants were infused several hours before the end of the light period, and harvested about 24 hours later. The culm infusion procedure was used to introduce the labeled sucrose into the normal plant distribution routes, thus time needed to be allowed for the labeled sucrose to be distributed throughout the plant. A 15-h period had allowed a good distribution of label throughout the plant in a previous study [19], while 24 h had also proved practical in another study [1]. The 24-h interval was used here to allow the distribution to take advantage of a full diurnal cycle and approach steady state. A distribution approaching steady state was desired when examining the compartmental path of the sucrose during radial transfer. After 24 h, tissue was sampled from a) mature culm below and opposite the site of infusion (to avoid sucrose introduced into xylem or transferred directly through culm tissue), and b) internodal portions of an axillary branch which was at or prior to anthesis. The harvested plant material was bagged and stored on ice about one hour until processed.

### Differential extraction of free-space and intracellular compartment

Once samples were brought to the laboratory, the infused internode was sliced longitudinally into quarters. The quarter opposite the site of infusion was sliced into 3-mm sections with a razor blade. The razor blade was previously wiped with ethanol to remove oils. The resulting tissue sections were pooled in groups of two according to distance below the height equivalent to that of the site of infusion. When sampling the axillary branches, the middle portion of the internode was similarly quartered and sliced, with a sufficient number of the slices taken to approximate the tissue amount of the mature-culm samples.

The soluble sugars were differentially extracted from free-space and intracellular compartments of each group of sections through the following procedure [7]. First, sugars were extracted from the free space. Each group (typically about 80-mg dry weight) was rinsed with 4-mL buffer (25 mM MES [2-(*N*-morpholino)ethanesulfonic acid], pH 5.5, 250 mM mannitol, 1 mM CaCl_2_) for 2 minutes at room temperature to remove free-space solutes. Rinses were repeated with fresh buffer: once for 6 minutes and once for 22 minutes. During these latter rinses, humidified air was bubbled through the solution [7]. At the completion of a rinse, solutions were plunged directly into ice-cold methanol (12 mL of pooled rinses and 36-mL methanol to make final 75% [v/v] [18.5 M] solution). Intracellular-compartment soluble sugars were removed by agitation of remaining tissue for 30 minutes at 65°C in 15-mL 75% methanol. This step was repeated once with fresh aqueous (75%) methanol. These extracts were pooled on ice. Subsamples were removed for long-term storage at -20°C.

### Radiolabel concentrations of soluble sugars

Free-space and intracellular-compartment extracts were treated identically during analyses.

Samples in 75% methanol were treated with 10- to 15-mg activated charcoal (Sigma C-5385) per mL to remove substances that might interfere with enzymatic analyses [21,37]. The slurry was stirred, and then allowed to settle overnight at -20°C. This process was repeated once before removing solution away from the settled charcoal. Methanol in solution was then evaporated off at 60°C.

The total radioactivity in each sample was determined by counting an aliquot using liquid scintillation spectroscopy (Beckman Instruments, Irvine, California, USA; Model LS7500). The calculations for determining the relative contributions of ^3^H and ^14^C with automatic quench correction were confirmed using simultaneous equations [38].

The aqueous solutions remaining after evaporation of methanol were brought to 9-mM K-acetate, pH 5.5, and final concentrations of 4.3 EU glucose oxidase (EC 1.1.3.4) (Sigma G-6891) per mL and at least 545 EU catalase (EC 1.11.1.6) (Sigma C-30) per mL, where EU (enzymatic unit) is defined as 1 μmol substrate converted per minute under the assay conditions used by the supplier. After 60 minutes at 37°C, 0.5 meq of the acetate form of Amberlite IRA-68 (Sigma), a weak anion exchanger, was added per mL. Samples were agitated at room temperature for two hours, and liquid removed off of the settled resin to be saved for additional analyses. An aliquot was counted to determine the radioactivity removed as enzymatically converted glucose and possibly as other anions. The water content of the remaining resin slurry was determined by oven-drying [14]. This step was necessary to be able to calculate the total volume from which the aliquot was removed for counting.

Fructose was the next sugar to be selectively removed from solution. The solution was brought to 5.5-mM Bis-Tris (bis [2-hydroxyethyl]imino-tris [hydroxymethyl]-methane; 2-bis [2-hydroxyethyl]-amino-2-[hydroxymethyl]-1,3-propanediol), pH 6.8. A hexokinase (EC 2.7.1.1) reaction was performed [14] in this buffer to completely convert fructose to a phosphate form for removal by strong anion exchange resin [14]. After this, solution was removed off of the resin and saved for additional analyses. An aliquot was counted to determine radioactivity removed to the resin as enzymatically converted fructose. The water content of the remaining resin slurry was again determined.

Water can become tritiated due to a solvent-exchange side reaction of phosphoglucoisomerase (EC 5.3.1.9) [39] or through complete oxidation of tritiated carbohydrates. An activated charcoal:powdered cellulose spin column was devised for rapid and quantitative removal of water from sucrose in multiple small samples [20]. A 1:1 (w/w) mixture of Darco G-60 activated charcoal (Aldrich Chemical Co., Milwaukee, Wisconsin, USA, catalog no. 24,227-6) and Sulka-Floc powdered cellulose (James River Corp., Hackensack, New Jersey, USA, SW-40 grade) was made. The column was prepared by pouring 400 mg of the mixture into a 5-mL disposable hypodermic syringe barrel. A glass microfiber disk was used to hold the powder in place. Before use, the column was washed by spin elutions: several times with 1-ml additions of water, several times with 75% methanol, and several more times with water. A spin was 1800 g for 1 minute at room temperature. The 1-mL aliquot of sample was applied, the column spun, and the first eluate collected. Four elutions, each of 1-mL water, then five elutions, each of 1-mL 80% methanol, followed. All ^3^H_2_O eluted in the first three eluates (mean = 99.8%; 95% c.i. = 97.1 – 102.5%); sucrose was recovered in the aqueous-methanol eluates (mean = 94.1%; 95% c.i. = 93.5 – 94.7%). Separation was complete (0.4% ^14^C in H_2_O eluates; 95% c.i. = -0.02 – 1.0%).

Sucrose was recovered in 80% methanol. To concentrate the solution as well as to move sucrose into aqueous solution for enzymatic analysis, methanol was evaporated off at 60°C. An aliquot was removed for counting to determine remaining radioactivity. The sucrose was then cleaved by addition of invertase (EC 3.2.1.26) (United States Biochemical Co., Cleveland, Ohio, USA; catalog no. 17676) at 2.9 EU per mL final concentration. The invertase supplemented the addition of buffered glucose oxidase and catalase that was otherwise identical to that described earlier for the enzymatic conversion of glucose. This reaction was allowed 90 minutes at 37°C. Other steps for determining radioactivity in the hexose moieties of sucrose were identical to those described earlier for determining amounts in glucose and fructose.

### Concentrations of specific soluble sugars

Glucose, fructose, and sucrose contents of the aqueous solution remaining after the initial evaporation of methanol were determined. These assays, which relied upon coupled-enzyme methods for stoichiometric production of NADH, were further coupled to allow stoichiometric reduction of INT (iodonitrotetrazolium violet). The absorbance of reduced INT product was read at 492 nm. The assays were performed in microtiter plates according to Hendrix [40], but with the modifications supplied by Tarpley et al. [14]. These modifications include the addition of albumin and detergent to help stabilize the chromophore in homemade reaction solutions.

### Statistical methods

The nature of the data suggested that a presentation of 95% confidence intervals (c.i.) about the mean was sufficient for interpretation. Confidence intervals are based on untransformed data. Transformations to account for proportional data and for non-normality did not alter any conclusions.

Randomization of label distribution between the two hexose moieties of sucrose was modeled as a Poisson process because the chance of an individual hexose molecule being involved in a particular catalysis event was rare [41].

## Authors' contributions

Culture of plants, development of sorghum as a model for whole-plant physiology study of photosynthate partitioning and allocation (DMV); development of the laboratory analytic procedures, conductance of the study, writing of the manuscript (LT). Both authors read and approved the final manuscript.
